# Experimental infection of purebred Saanen goats high pathogenicity and virulence of *Babesia aktasi*

**DOI:** 10.1371/journal.pntd.0012705

**Published:** 2024-12-02

**Authors:** Mehmet Can Ulucesme, Sezayi Ozubek, Munir Aktas

**Affiliations:** Department of Parasitology, Faculty of Veterinary Medicine, University of Fırat, Elazig, Turkey; Center for Biologics Evaluation and Research, Food and Drug Administration, UNITED STATES OF AMERICA

## Abstract

Small ruminant babesiosis remains a neglected disease despite causing significant economic losses to sheep and goat herds in many regions around the world. The pathogenesis and clinical manifestations of ovine babesiosis are well-known, but there is a lack of information regarding caprine babesiosis. Since the discovery of the first *Babesia* spp. in 1888, several species/subspecies/genotypes, including *Babesia aktasi*, have been described. Our recent molecular survey revealed that the parasite is highly prevalent (22.5%) in indigenous goats from Mediterranean region of Türkiye. The aim of this experimental study was to determine the pathogenicity and virulence of *B*. *aktasi* in immunosuppressed (n = 5) and immunocompetent (n = 7) purebred Saanen goats. The goats were experimentally infected with fresh *B*. *aktasi* infected blood, and examined for clinical, parasitological, hematological, and serum biochemical findings throughout the infection. Following the parasite inoculation, intra-erythrocytic parasites were detected from the 1st day post-infection, followed by an increase in rectal temperature and parasitemia. The parasitemia was detected ranging from 4.3% to 33.5% in the immunosuppressed group, while it was 2.1% to 7.6% in the immunocompetent. Severe clinical symptoms characterized by anemia, jaundice, and hemoglobinuria developed in both groups. A statistically significant inverse correlation was observed between the increase in parasitemia and RBC, WBC, HCT, and Hb values in the goats compared to pre-infection levels. Values of AST, ALT, GGT, Total bilirubin, and Albumin showed a significant increase, with all the immunosuppressed goats dying on the 4^th^ and 7^th^ days post-infection, while four out of seven immunocompetent goats died on between 6-8^th^ days. Severe edema in the lungs, frothy fluid in the trachea, jaundice in the subcutaneous and mesenteric fat, and dark red urine were detected in necropsy. The results obtained in this study demonstrated that *B*. *aktasi* was highly pathogenic to purebred Saanen goats. Current work assures valuable insights into the pathogenesis and virulence of *B*. *aktasi* and serves as a foundation for future studies to develop effective control strategies against caprine babesiosis.

## Author summary

Advancements in molecular parasitology have led to the discovery of novel *Babesia* species/genotypes, including *Babesia aktasi*, identified in goats in the Mediterranean region of Türkiye. Host resistance against blood protozoa such as *Babesia* and *Theileria* exhibits different behaviors, but there is a lack of information about host resistance against *Babesia* spp. in goats and sheep. The pathogenicity of *B*. *aktasi* in local breeds has been revealed in previous studies. There may be differences in susceptibility to *Babesia* parasites between different breeds of the same host species. To better understand the biology of *B*. *aktasi*, its pathogenesis and virulence were revealed by experimental infection in immunosuppressed and immunocompetent purebred Saanen goats. Saanen goats in both groups were infected with fresh blood infected with *B*. *aktasi* and a rapid increase in parasitemia was observed. Both groups experienced severe clinical infection, resulting in mortality. Significant changes in hematological and biochemical parameters compared to pre-infection levels were noted in both groups, revealing that *B*. *aktasi* is highly pathogenic for purebred Saanen goats. It is a step forward in elucidating the biology of *B*. *aktasi* and provides a basis for future studies to develop effective control strategies against caprine babesiosis.

## Introduction

*Babesia* is a genus of intra-erythrocytic protozoan parasites in various parts of the world, where the responsible vector ticks are prevalent [1–3]. Since the first discovery of *Babesia* in 1888, many species, subspecies or genotypes have been described that infect domestic and wild mammals, some of which also have zoonotic character [4]. Typically, *Babesia* parasites cause serious clinical signs characterized by high fever, hemolytic anemia (destruction of red blood cells), icterus, hemoglobinuria, and in severe cases, death [5]. *Babesia ovis*, *B*. *motasi* and *B*. *crassa* are the main *Babesia* spp. responsible for small ruminant babesiosis [3,6,7]. Of these, *B*. *ovis* is one of the first identified *Babesia* spp. caused by clinical infection with high mortality in tropical and subtropical countries [8–10].

In light of advances in molecular parasitology in recent years, novel species or genotypes have been added to the blood protozoans belonging to the *Babesia* genus. In this context, *Babesia lengau*-like, *Babesia* sp. Xinjiang and *B*. *motasi*-like complex have been reported to cause babesiosis in small ruminants [6,11–13]. Studies based on apicoplast and mitochondrial genome analyses revealed that the parasites in the *B*. *motasi*-like group were divided into two different branches, one branch includes *Babesia* sp. Lintan and *Babesia* sp. Tianzhu, while the other includes *Babesia* sp. Hebei and *Babesia* sp. Ningxian [14,15]. In a recent study based on molecular methods, we have identified a novel *Babesia* sp. that is distinct from other *Babesia* pathogens [16]. According to Robert Koch’s postulates [17], then, *in vivo* isolation of the newly identified *Babesia* sp. was made from a naturally infected goat, and named *Babesia aktasi* [18]. In our large molecular survey carried out in the Mediterranean region of Türkiye, it was detected that 22.5% of the goats (113/503) were infected with the parasite [19]. Then, an experimental study performed on the immunocompetent goats revealed that *B*. *aktasi* did not cause typical clinical findings of babesiosis (anemia, icterus, hemoglobinuria) except for increased body temperature [20].

Türkiye serves as a vast land bridge between European and Asian countries, where ovine babesiosis is significant due to the favorable geographical and climatic conditions that support the maintenance of vector ticks [21]. This disease is the most common parasitic infection that seriously affects the health of sheep and goats in Türkiye, leading to substantial economic losses because of the costs associated with control and treatment [7,9]. The recent nationwide epidemiological study conducted in Türkiye indicates that the distribution and prevalence of ovine babesiosis can vary regionally [22]. In particular, the disease is reported to be more prevalent in the Central and Southeastern Anatolia regions of Türkiye [22]. *Babesia ovis*, *B*. *crassa*, and *B*. *motasi* have been molecularly identified in small ruminant and ixodid ticks in Türkiye [19,23–26].

Host resistance to blood protozoans such as *Babesia* and *Theileria* involves a complex interplay of various immune responses [27,28]. The host resistance to these parasites can vary among different host species and even among individuals within a species [29]. The pathogenicity of these parasites is expected to be higher in purebred breeds than in native breeds. It has been reported that purebred *Bos indicus* cattle show higher levels of resistance to babesiosis than *Bos taurus* [30]. The pathogenicity of *B*. *aktasi* in purebred Saanen goats is unknown. It is important to conduct pathogenicity studies to comprehensively understand the ability of *B*. *aktasi* to cause clinical disease in purebred goats. Therefore, an experimental study using fresh *B*. *aktasi*-infected blood was performed to assess pathogenicity and virulence of the parasite in both immunosuppressed and immunocompetent purebred Saanen goats.

## Materials and methods

### Ethics statement

This study was approved by the Fırat University Animal Experiments Local Ethics Committee (session number: 2021/12).

### Research material

The stabilate of *B*. *aktasi*, as previously described by Ozubek et al. [20], was used in this study. An indigenous goat was experimentally infected with the stabilate. When parasitemia (PPE) reached 9.2–11.5%, jugular venous blood was collected into vacutainer tubes coated with EDTA. Infected red blood cells were cryopreserved with 10% dimethyl sulfoxide (DMSO) in 5 mL aliquots and frozen in liquid nitrogen. In this study, these batches of stabilates were used for the experimental infection of indigenous donors to obtain fresh blood infected with *B*. *aktasi*.

### Selection of purebred Saanen goats for experimental infection

The sample size required to detect a significant difference between two independent groups was determined through a priori power analysis using the G*Power program (Version 3.1.9.3) before starting the experiments [31]. A one-tailed t-test was selected with an effect size (d) of 1.5 and a significance level (α) of 0.05. The required sample size was determined to be 7 animals per group, for a total of 14 animals. The actual power achieved with this sample size was 0.841.

**Fig 1:**
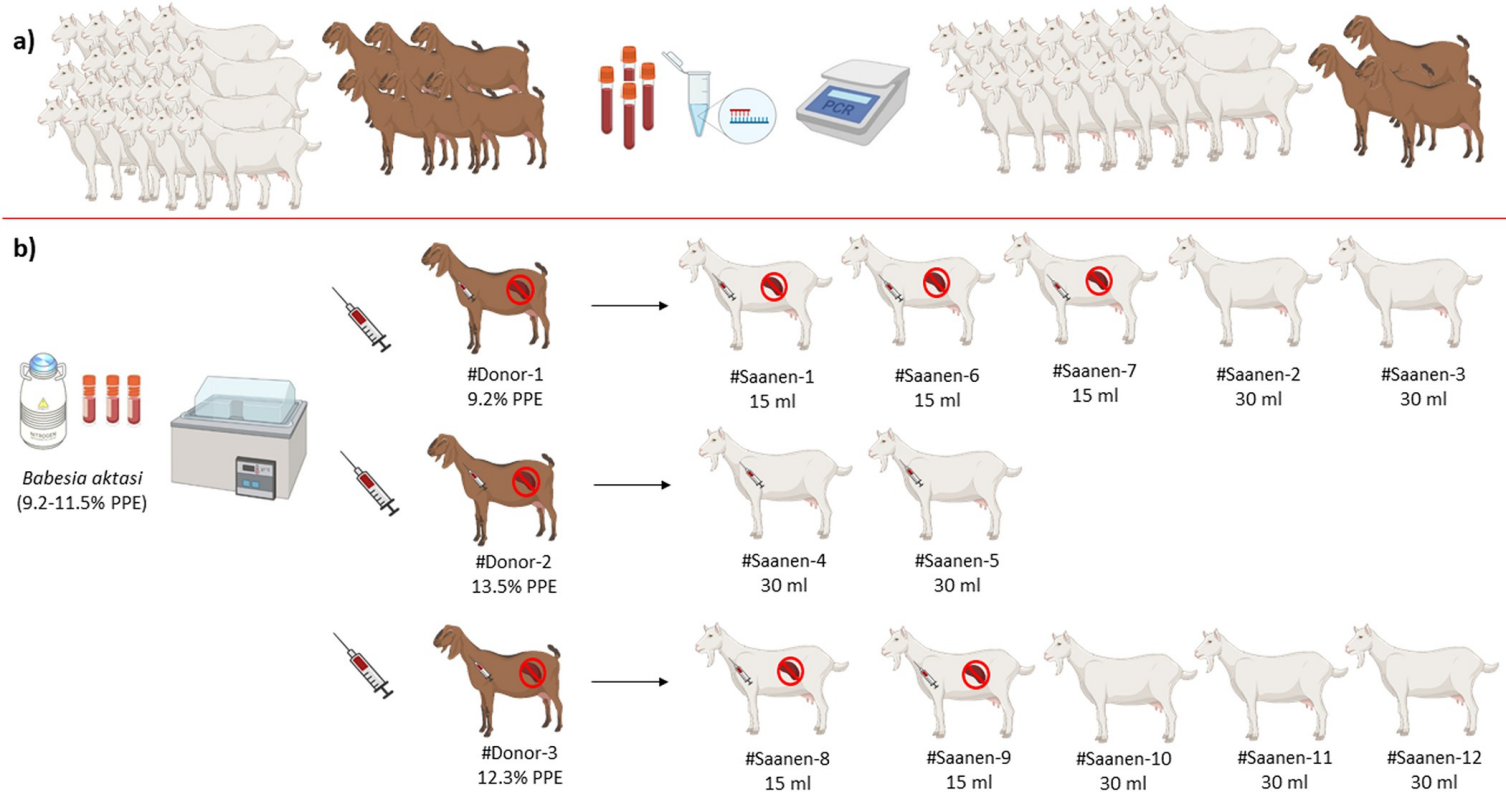
Representative diagram of experimental design. a) Selection of tick-borne pathogen-free (*Babesia* spp. *Theileria* spp., *Anaplasma* spp.) purebred Saanen and indigenous goats; b) Infection of the immunosuppressed indigenous donors with *B*. *aktasi* stabilates (PPE 9.2–11.5%) to obtain the fresh infected blood for the infection of immunosuppressed and immunocompetent Saanen goats. Fig 1 was created with BioRender.com (www.biorender.com).

The representative design of the experimental infection conducted in this study is shown in Fig 1.

Prior to experimental infection, 5 mL of whole blood were collected in vacutainer tubes containing EDTA from 5-month-old purebred Saanen (n = 22) and indigenous male goats (n = 15) from *B*. *aktasi*-free breeding farms in Elazig province. A small amount of peripheral blood was taken from the ear vein of each goat using a sterile needle to prepare thin blood smears. The samples were brought to the laboratory at the Fırat University Faculty of Veterinary Medicine, Department of Parasitology. Thin blood smears and DNA isolated from EDTA blood samples were screened for the presence of *Babesia* spp., *Theileria* spp., and *Anaplasma* spp. using microscopy and nested PCR [19,24]. Seventeen male goats (14 purebred Saanen, 3 indigenous goats) that tested negative for the selected pathogens were purchased and maintained at the Ministry of Agriculture, Elazığ Veterinary Control Institute, small ruminant unit. It was ensured that the goats were of similar age, weight, and sex to minimize variables affecting on the results of experimental study. Each of the goats maintained at the unit were raised in a separate tick-free pen, and kept in quarantine for 3 weeks. In order to eliminate tick exposure, the goats were treated with Flumethrin 1% pour-on (Ba-tick, BaVET, 10 mg Flumetrin, 100 mL, Türkiye) every 21 days during the study. Following the quarantine period, microscopy and nested PCR were repeated, and it was confirmed that they were free of *Babesia* spp., *Theileria* spp., and *Anaplasma* spp.

### Splenectomy and post-operative care

In this study, a randomized controlled experimental infections were performed with two groups of purebred Saanen goats, one consisting of immunosuppressed (splenectomy + dexamethasone administered) goats (n = 7) and the other consisting of immunocompetent (spleen-intact + not dexamethasone applied) individuals (n = 7). The goats in the immunosuppressed group underwent splenectomy using standard anesthesia, analgesic and surgical techniques at Fırat University Animal Hospital before the experimental infection [20,32]. Splenectomized goats were maintained in separate pens throughout the experiment. As post-operative care, the goats were stabilized by administering intravenous 0.9% physiological saline (250 mL) and 5% dextrose (250 mL) once a day for 2 days. In order to prevent possible bacterial infections, a 5-day course of intramuscular antibiotic treatment was administered after splenectomy. Additionally, antibiotic spray (Neocaf, MSD, Oxytetracycline, aerosol spray suspension, 200 mL, USA) was applied to the surgical wound every day for a week. The rectal temperature and clinical symptoms were monitored every two days. The goats were also examined by microscopy and nested PCR for the presence of *Babesia* spp., *Theileria* spp. and *Anaplasma* spp. twice a week.

### Experimental infection of immunosuppressed indigenous goats with *B*. *aktasi* stabilate

In our previous study, we demonstrated that the parasite has a high level of pathogenic effect in immunosuppressed indigenous goats [20]. Therefore, an immunosuppressed indigenous goats were used as donors to obtain a source of fresh blood infected with the parasite in this study. As seen in Fig 1, the *B*. *aktasi* stabilates with 9.2–11.5% PPE kept in the liquid nitrogen was thawed at 37°C, and 15 mL was intravenously inoculated to the donor goats (#Donor-1, #Donor-2, and #Donor-3). Following the parasite inoculation, dexamethasone was also intramuscularly injected to the donors as previously described [11,20]. Subsequent, the donors were monitored daily for the clinical and parasitological findings. When the PPE reached approximately 9.2% and 13.5% on the 12^th^ day post-infection (DPI) in #Donor-1 and #Donor-2, respectively, and 12.3% on the 8^th^ DPI in #Donor-3, venous blood was collected from the donors, and utilized for the experimental infection of the Saanen goats. Then, the donors received treatment with imidocarb dipropionate (0.1 mg/kg body weight IM, İmicarp, Teknovet, Türkiye) and oxytetracycline (10 mg/kg bodyweight IM once daily for 5 days, Primamycin/LA, Zoetis, USA).

### Experimental infection of purebred Saanen goats with fresh blood infected with *B*. *aktasi*

Three immunosuppressed Saanen goats (#Saanen-1, #Saanen-6 and #Saanen-7), and two immunocompetent (#Saanen-2, and #Saanen-3) were injected intravenously with 15 mL and 30 mL, respectively, of fresh blood infected with *B*. *aktasi* from the indigenous #Donor-1 (PPE 9.2%). Two immunocompetent Saanen goats (#Saanen-4, #Saanen-5) were each injected with 30 mL of fresh infected blood from the #Donor-2 (PPE 13.5%) (Fig 1). Similarly, two immunosuppressed Saanen goats (#Saanen-8 and #Saanen-9) and three immunocompetent Saanen goats (#Saanen-10, #Saanen-11, and #Saanen-12) were injected with 15 mL and 30 mL, respectively, of fresh blood infected with *B*. *aktasi* from #Donor-3 (PPE 12.3%) (Fig 1). Post-injection of the infected blood, rectal temperature, general physical condition and the presence of specific clinical signs of babesiosis (anemia, jaundice, and hemoglobinuria) were assessed daily for the duration of the experimental infections. Additionally, peripheral blood smears from the goats were daily prepared for microscopic examination. Blood samples from the jugular venous of each goat were also collected in EDTA and clot activator vacutainer tubes. The anticoagulated blood samples were utilized for hematological analysis and DNA extraction, while the sera were used for serum biochemical profiles.

### Microscopic examination of peripheral blood smears

Thin blood smears from the peripheral blood were fixed with absolute methanol (Scharlau, Spain) for 5 minutes, then allowed to dry and stained with 10% Giemsa solution (Carlo Erba Reagents, France) for 30 minutes. The stained blood smears prepared from the goats were examined for the presence of intra-erythrocytic stages of *Babesia* spp., *Theileria* spp. and *Anaplasma* spp. under BX43 light microscopy (Olympus, Tokyo, Japan) with 100X objective (x1000 magnification). Twenty microscope fields randomly selected from each of blood smear were scanned, and PPE was calculated as previously reported [20,32].

### DNA isolation and Polymerase Chain Reaction (PCR)

Genomic DNA was extracted from 200 μl whole-blood samples using a commercial DNA isolation kit (PureLink Genomic DNA Mini Kit, Invitrogen Corporation, Carlsbad, USA) according to the manufacturer’s instructions. The DNA was stored at -20°C until PCR amplification. *Babesia*/*Theileria* [33,34], and *Anaplasma* [35,36] genus specific nested PCR protocols were used to amplify *18S rRNA* and *16S rRNA* genes, respectively. The PCR reaction and thermal cycling conditions were as described by Ozubek et al. [20]. In each PCR mixture, reference positive control DNAs for *B*. *aktasi* (OQ120434), *T*. *ovis* (EF092452), *B*. *ovis* (EF092454) and *A*. *ovis* (MG693754) available in our laboratory stocks and previously confirmed by sequencing were used. Distilled water and genomic DNA isolated from *Babesia* spp., *Theileria* spp. and *Anaplasma* spp. free 1-month-old goat was used as negative controls. Ten μl of the PCR products were run on 1.4% agarose gel for 30 minutes. After electrophoresis, the agarose gel was stained with Ethidium Bromide (Sigma-Aldrich, USA) for 20 minutes and examined on the Quantum Vilber Lourmat (France) gel imaging system.

### Hematology and serum biochemistry

Hemogram and serum biochemistry analyzes were performed at before the experiment and from appearance to disappearance of intracellular parasites in blood smears. The following parameters were examined for hematological analysis: Red blood cell count (RBC), hemoglobin level (Hb), hematocrit level (HCT), white blood cell count (WBC), mean corpuscular volume (MCV), mean corpuscular hemoglobin concentration (MCHC), red cell distribution width-coefficient of variation (RDW-CV), and red cell distribution width-standard deviation (RDW-SD). The separated serum samples were analysed for estimation of biochemical parameters of Creatinine, Total protein, Albumin, Total bilirubin, Glucose, Alanine aminotransferase (ALT), Aspartate aminotransferase (AST), and Gamma glutamyl aminotransferase (GGT). The hematology and serum biochemistry analyses were conducted at Fırat University Animal Hospital using Mindray BC-5000 Vet and NX500 Auto Haematology Serum Biyochemistry Analysers (Bio-Medical Electronics Co. Ltd., Shenzhen, China), respectively. Hematobiochemical changes in the experimentally infected goats were compared with pre-infection levels.

### Statistical analysis

A comparison of hemogram and serum biochemistry values was conducted between the immunosuppressed and immunocompetent Saanen goats. The SPSS package program (IBM SPSS Statistics Version 21) was used for statistical analyses. The appropriateness of the data for parametric or non-parametric tests was assessed using the Shapiro-Wilk test, while the homogeneity of variances was evaluated using the Bartlett test. For parameters subjected to pairwise comparisons and exhibiting a normal distribution, independent samples Student t-test was employed, whereas dependent samples t-test was used for dependent variables. Statistical analyses were conducted using the Mann-Whitney U test for samples not conforming to normal distribution. Data were presented as mean and standard error. The statistical significance level was set at *p<*0.05.

## Results

### Pathogenicity of *B*. *aktasi* in immunosuppressed Saanen goats

**Fig 2:**
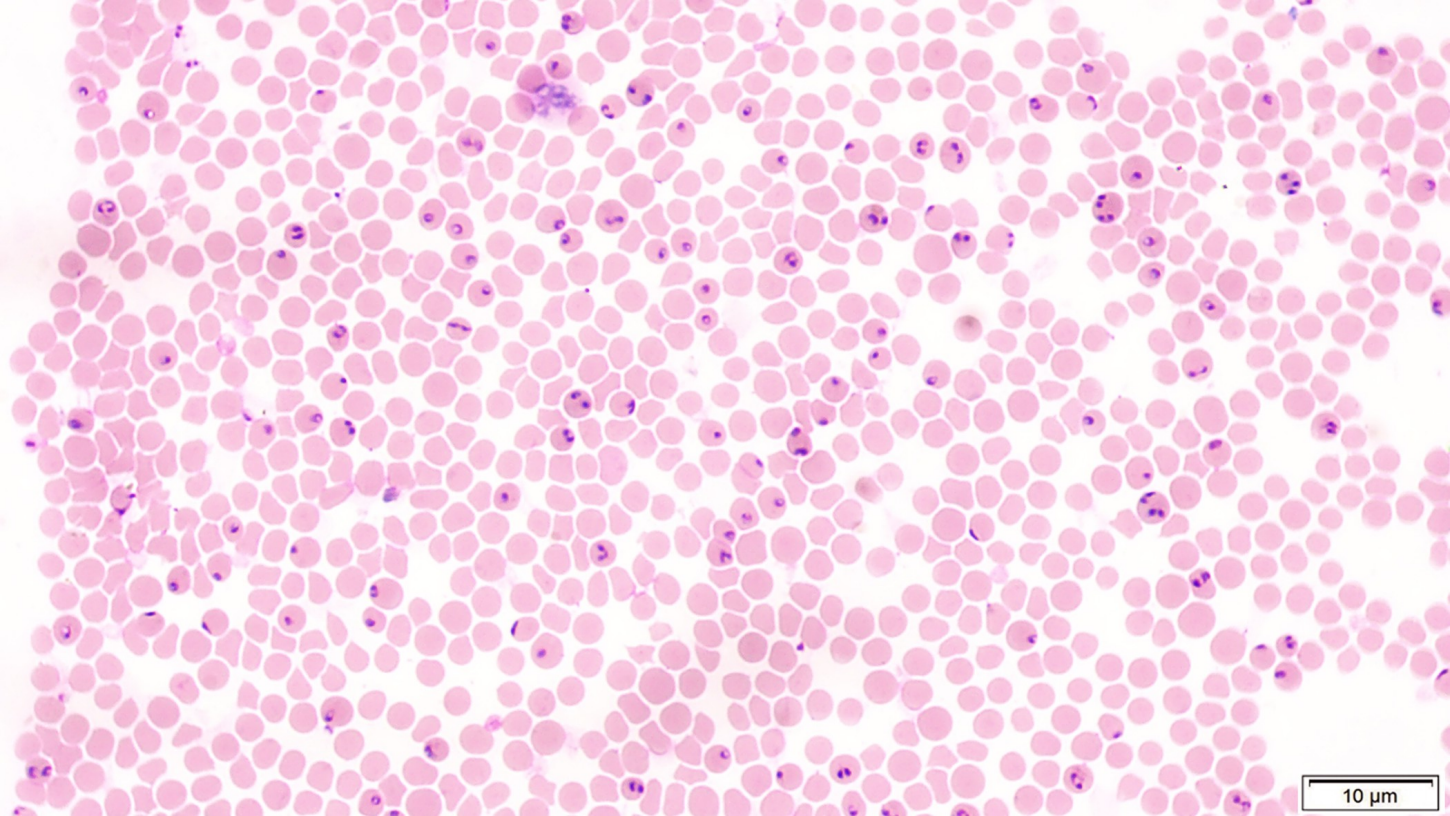
Intra-erythrocytic stages of *B*. *aktasi* in a blood smear from an immunosuppressed Saanen goat (Animal ID. #Saanen-1).

After the injection of fresh blood infected with *B*. *aktasi*, all immunosuppressed goats developed severe clinical infection. Correlated with the appearance of intracellular parasites, an increase in rectal temperature up to 42.2°C was measured in the goats. Characteristic signs of clinical disease (anemia, jaundice and evident hemoglobinuria) were detected in all Saanen goats, however, it is noteworthy that the signs of anemia and jaundice were mild. In addition to these specific findings, the Saanen goats also exhibited general clinical signs such as lethargy, loss of appetite, teeth grinding, trembling, moaning, rapid breathing, immobility, and leaning their heads on the ground. The infection progressed rapidly, and all immunosuppressed Saanen goats died on the 4^th^ day post-inoculation, except for one (#Saanen-9) that died on the 7^th^ day. High PPE levels, ranging from 4.3% to 33.5%, were detected in the blood smears (Fig 2). Seven Saanen goats were initially selected for the immunosuppressed group. However, four goats (#Saanen-1, #Saanen-6, #Saanen-7, and #Saanen-8) died on the 4^th^ day post-infection (DPI), and one goat (#Saanen-9) died on the 7^th^ DPI due to severe clinical signs of babesiosis (Table 1). Following the deaths of these five animals, the remaining two goats were not experimentally infected for ethical reasons. This decision was made to minimize the number of animals used in accordance with ethical guidelines, while ensuring that sufficient data were obtained to address the study objectives.

**Table 1:** Source infection and PPE, amount of inoculation and route, changes in infection parameters, and the infection outcome in immunosuppressed and immunocompetent Saanen goats (*died due to severe acute illness caused by *B*. *aktasi*).

**Immunosuppressed goats**	**Source of infection and PPE (%)**	**Inoculation amount and route**	**Prepatent period (days)**	**Max. PPE (%)**	**Max. fever (** ^**o**^ **C)**	**Duration of fever days**	**Died DPI**
#Saanen-1*	Fresh blood (9.2%)	15 mL (iv)	1	33.5	42.1	2	4
#Saanen-6*	Fresh blood (9.2%)	15 mL (iv)	1	15	41.4	2	4
#Saanen-7*	Fresh blood (9.2%)	15 mL (iv)	1	21.2	42.2	3	4
#Saanen-8*	Fresh blood (12.3%)	15 mL (iv)	1	12.5	41.5	2	4
#Saanen-9*	Fresh blood (12.3%)	15 mL (iv)	1	4.3	41.7	6	7
**Immunocompetent goats**							
#Saanen-2	Fresh blood (9.2%)	30 mL (iv)	1	2.1	41.6	8	-
#Saanen-3	Fresh blood (9.2%)	30 mL (iv)	1	4.3	41.7	3	-
#Saanen-4*	Fresh blood (13.5%)	30 mL (iv)	1	7.6	41.7	8	8
#Saanen-5*	Fresh blood (13.5%)	30 mL (iv)	1	5.2	41.6	8	8
#Saanen-10*	Fresh blood (12.3%)	30 mL (iv)	1	3.6	41.8	6	6
#Saanen-11*	Fresh blood (12.3%)	30 mL (iv)	1	3.7	41.4	6	7
#Saanen-12	Fresh blood (12.3%)	30 mL (iv)	1	2.5	41.2	6	-

### High Virulence of *B*. *aktasi* to immunocompetent Saanen goats

After the inoculation, serious clinical infections resulting in death occurred in the immunocompetent Saanen goats. With the appearance of the intracellular parasite, an increase in rectal temperature up to 41.8°C was measured (Table 1). Specific clinical signs of babesiosis (anemia, jaundice, and hemoglobinuria) were observed in all the immunocompetent Saanen goats. Additionally, symptoms such as lethargy, loss of appetite, teeth grinding, tremors, rapid breathing, and inability to stand were observed in the goats. In the #Saanen-2, #Saanen-3, and #Saanen-12, improvement in clinical symptoms was observed along with the disappearance of parasites from peripheral blood on the 6^th^ and 7^th^ DPI, and the goats survived the acute disease. However, #Saanen-10 died on the 6^th^ post-infection, followed by #Saanen-11 on the 7^th^, and both #Saanen-4 and #Saanen-5 on the 8^th^ day, due to severe clinical infection. Peak PPE, ranging from 2.1% to 7.6%, was detected on the 3^th^ DPI (Table 1).

### Hematological and serum biochemical findings

The pre-infection and post-infection hematological and serum biochemical values in immunosuppressed and immunocompetent Saanen goats are summarized in Table 2.

**Table 2:** Hematological and serum biochemical profiles between pre-infection and post-infection in immunosuppressed and immunocompetent Saanen goats. Statistical significance is indicated as follows: **** *p*<0.0001, *** *p*<0.001, ** *p*<0.01, * *p*<0.05.

	Immunosuppressed goats n = 5		Immunocompetent n = 7	
Hematology	Pre-infection	Post-infection	Pre-infection	Post-infection
RBC (x10^6^/μl)	22.94±1.96	15.36±2.65***	19.09±1.32	6.69±0.72****
WBC (x10^3^/μl)	21.27±0.99	9.81±2.30***	9.54±1.87	3.43±1.38****
HCT (%)	31.36±3.51	22.28±5.16**	29.03±1.35	11.40±1.42****
Hb (g/dl)	12.82±2.26	9.28±1.54*	10.71±0.63	3.79±0.43****
MCV (fL)	15.05±0.17	15.98±0.67	15.51±1.50	18.89±3.43*
MCH (pg)	5.50±0.25	5.90±0.37	5.44±0.54	6.21±0.79
MCHC (%)	38.10±1.41	37.47±0.31	36.70±1.45	32.01±1.82***
RDW-CV (%)	26.54±1.67	28.52±2.09	24.11±1.83	48.07±9.86***
RDW-SD (fL)	17.36±1.12	19.46±1.69*	16.20±0.68	42.97±11.22***
**Serum biochemistry**				
Glucose (mg/dL)	52.20±28.03	25.82±5.36	39.29±16.56	32.29±21.21
AST (U/L)	118.82±55.57	622.40±422.35*	77.14±12.39	275.86±80.65****
ALT (U/L)	17.41±7.77	66.84±40.08*	14.29±3.99	32.57±7.87***
GGT (U/L)	61.40±6.88	90.80±11.12**	50.14±16.00	74.43±28.40*
Total Bilirubin (mg/dL)	0.11±0.02	0.48±0.19**	0.11±0.03	1.07±1.15**
Total Protein (g/dL)	7.10±0.33	6.60±0.61	6.81±0.66	6.01±0.71*
Albumin (g/dL)	3.56±0.35	3.14±0.18*	3.34±0.24	2.91±0.15**
Creatinine (mg/dL)	0.80±0.20	0.75±0.16	0.67±0.24	1.09±0.35*

**Fig 3:**
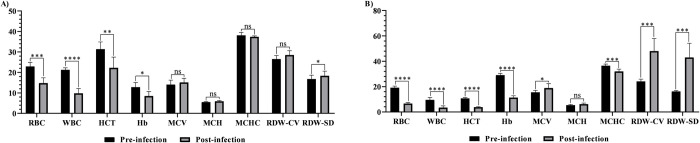
Comparison of hematological values between pre-infection and post-infection in immunosuppressed (A) and immunocompetent (B) Saanen goats. Statistical significance is indicated as follows: **** *p*<0.0001, *** *p*<0.001, ** *p*<0.01, * *p*<0.05, ns: not significant.

Significant changes in hematological parameters were observed in both experimental groups compared to pre-infection levels (Table 2 and Fig 3). The increase or decrease in hematological values was associated with the rise in PPE. A statistically significant decrease in RBC, WBC, HCT, and Hb values was observed in the both Saanen groups. An increase in MCV value was not statistically significant in immunosuppressed goats, whereas in the immunocompetent group was found to be significant. MCH value showed non-significant increase in the both groups. In the immunosuppressed group, a non-significant changes in MCHC and RDW-CV values was detected while significant increase was found in the immunocompetent goats. RDV-SD value showed significant increase in the both groups (Table 2 and Fig 3).

**Fig 4:**
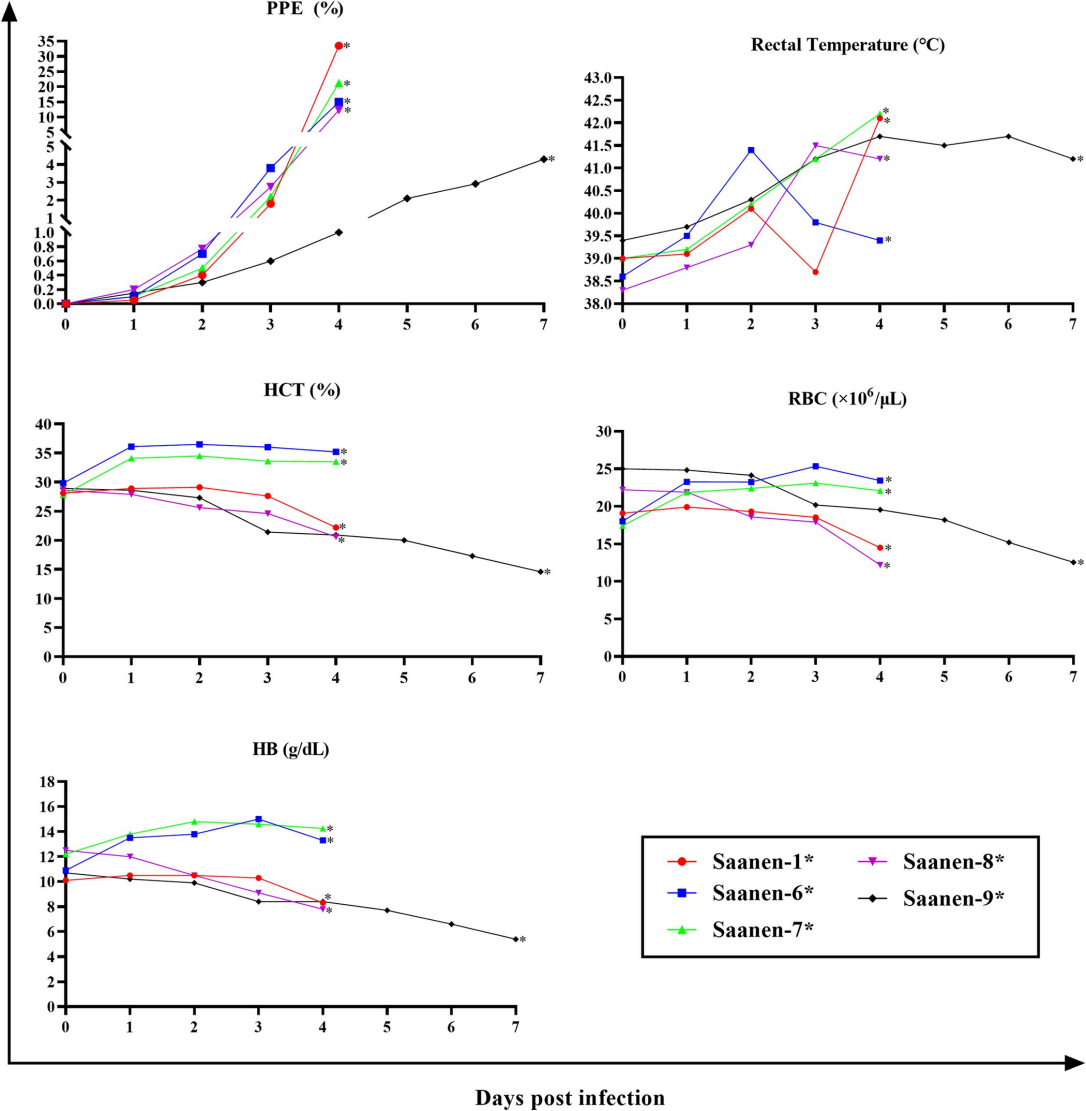
The course of PPE (%), rectal temperature (°C), Hb (g/dL), RBC (×10^6^/μL) and HCT (%) changes during acute infection in immunosuppressed Saanen goats infected with *B*. *aktasi* (*Saanen goats died due to severe clinical babesiosis caused by the *B*. *aktasi*).

**Fig 5:**
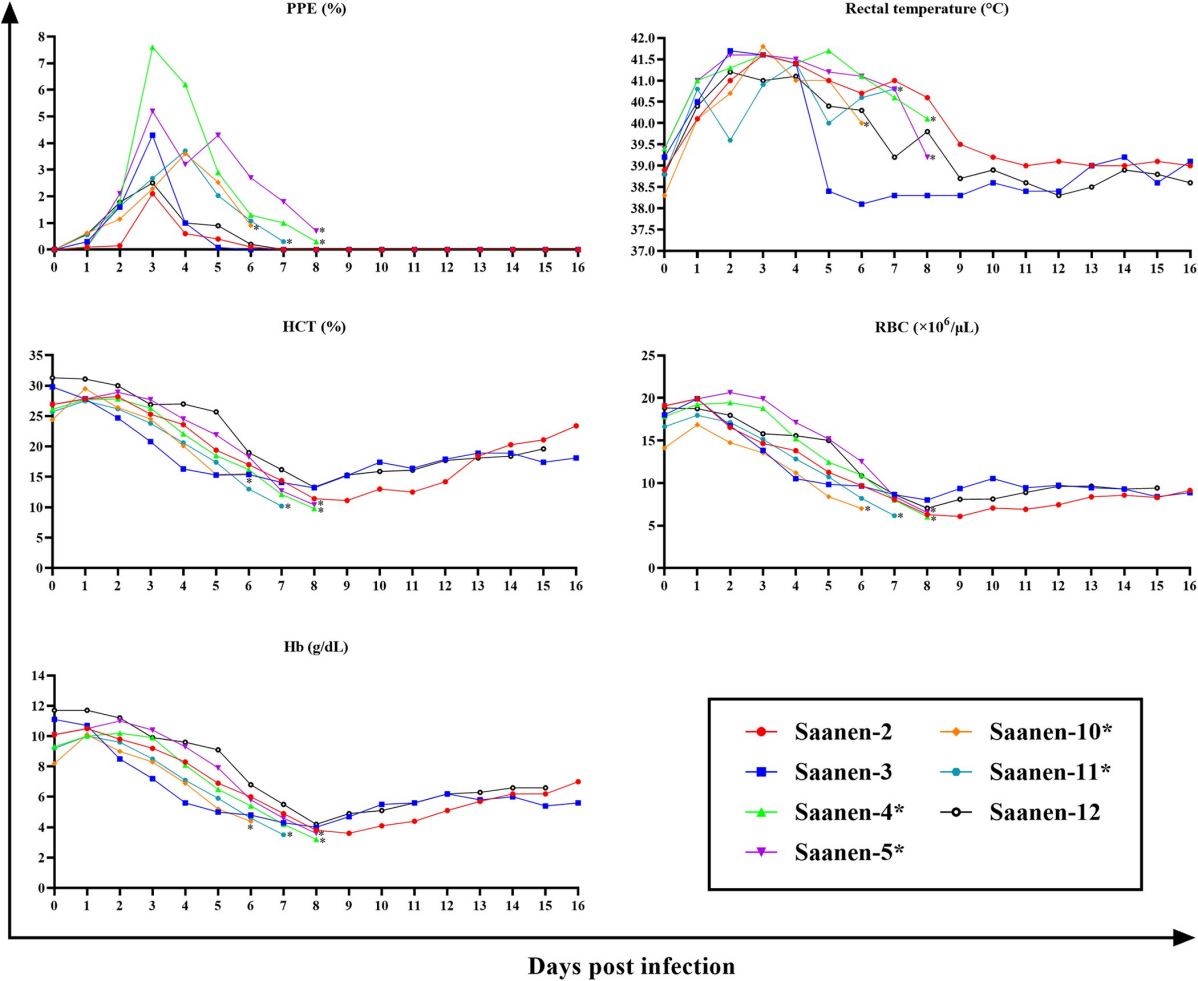
The progression of PPE (%), rectal temperature (°C), Hb (g/dL), RBC (×10^6^/μL), and HCT (%) during acute infection in immunocompetent Saanen goats infected with *B*. *aktasi* (*Saanen goats died due to severe clinical babesiosis caused by the *B*. *aktasi*).

Intra-erythrocytic stages of the parasite were first detected from the 1^st^ DPI in all experimentally infected Saanen goats (Table 1 and Figs 4 and 5). An increase in rectal temperature ranging from 41.2°C to 42.2°C was observed in correlation with the appearance of the parasites. In all of the immunosuppressed Saanen goats, the peak PPE was observed on the day of death, while it was observed within 4 days following parasite inoculation in the immunocompetent goats. In both groups, a decrease in HCT, RBC, and Hb values was observed as PPE increased. However, as the infection duration was longer in immunocompetent goats, the decrease in HCT, RBC, and Hb values reached their lowest levels when PPE decreased to a level almost undetectable by light microscopy (Fig 5). In the goats that survived the acute disease (#Saanen-2, #Saanen-3, and #Saanen-12), an increase in HCT, RBC, and Hb values began from the 8^th^ and 9^th^ days after parasite inoculation. Due to the death of other goats, an increases in these values (HCT, RBC, Hb) could not be determined. (Figs 4 and 5).

**Fig 6:**
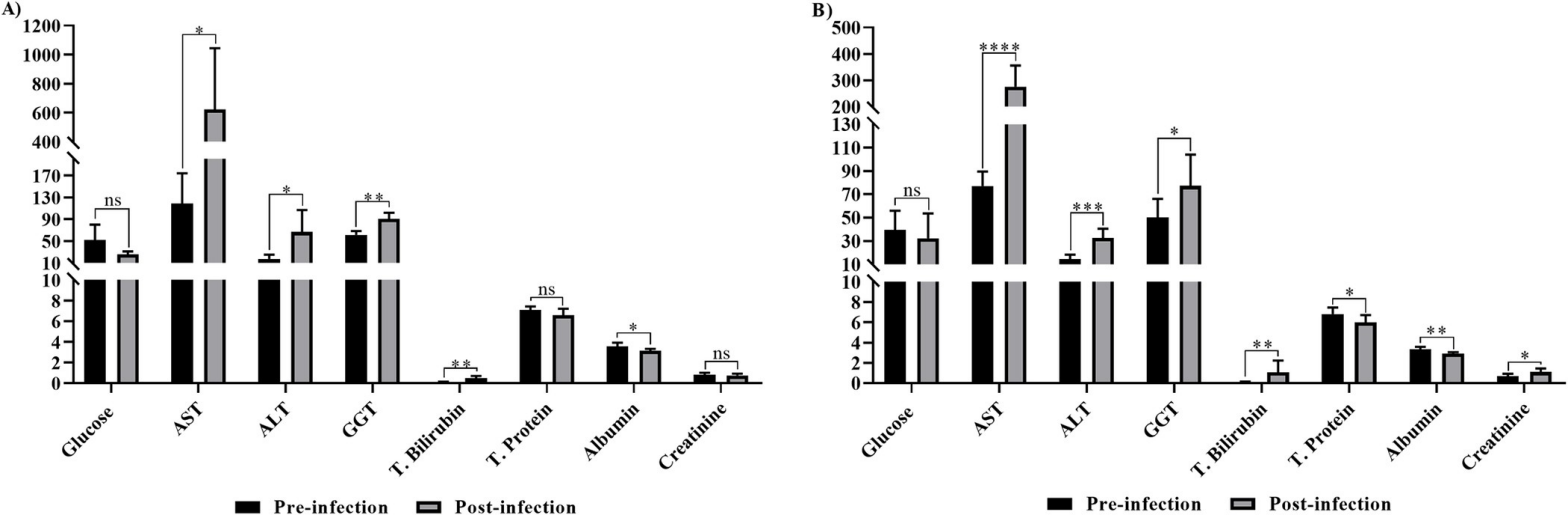
Comparison of serum biochemical profiles between pre-infection and post-infection in immunosuppressed (A) and immunocompetent (B) Saanen goats. Statistical significance is indicated as follows: **** *p*<0.0001, *** *p*<0.001, ** *p*<0.01, * *p*<0.05, ns: not significant).

There was a statistically significant increase in AST, ALT, GGT and total bilirubin values in the both infected groups compared to their pre-infection levels. However, albumin value exhibited a statistically significant reduction in the both infected groups. The decrease in total protein value and the increase in creatinine value were not significant in the immunosuppressed group, whereas but these changes were statistically significant in the immunocompetent group. The differences in glucose was not significant for any group (Table 2 and Fig 6).

### Necropsy

**Fig 7:**
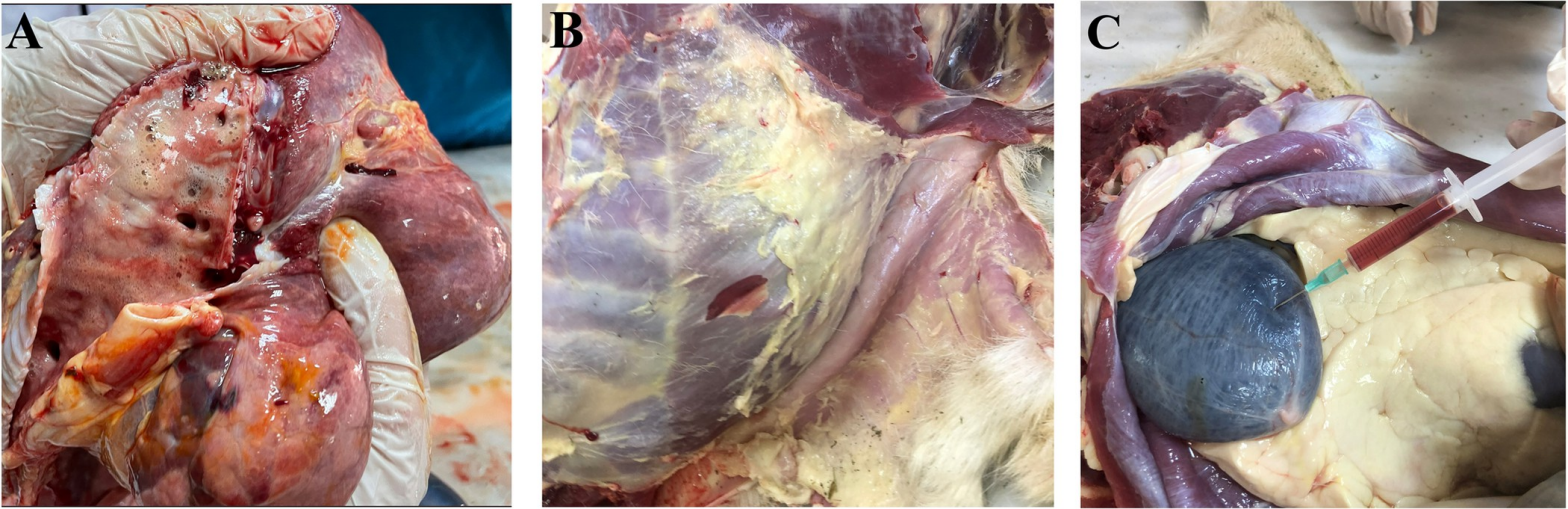
The macroscopic lesions in purebred Saanen goats experimentally infected with *B*. *aktasi*. A, pulmonary edema, frothy fluid in the lung and trachea; B, jaundice in subcutaneous and mesenteric adipose tissue; C, dark red urine in the bladder (hemoglobinuria).

Three out of 5 dead goats (#Saanen-1, #Saanen-4, and #Saanen-5) were submitted for necropsy. During necropsy, severe pneumonia associated with edema in the lungs, accumulations of frothy exudate in the trachea, jaundice in subcutaneous and mesenteric adipose tissue and dark red urine (hemoglobinuria) in the urinary bladder were detected in the dead goats (Fig 7A, 7B and 7C).

## Discussion

Small ruminant babesiosis causes substantial economic losses in sheep and goat herds worldwide and remains classified as a neglected disease [3,32,33]. The primary causative agents of small ruminant babesiosis include *B*. *ovis*, *B*. *motasi*, and *B*. *crassa* [4]. One of the initially identified *Babesia* spp., *B*. *ovis* has been reported to cause clinical infections with high mortality in sheep under field conditions in the Southern Europe, Middle East, African and Asian countries [9,37–39]. *Babesia crassa* is non-pathogenic in sheep and goats, whereas *B*. *motasi* is considered moderately pathogenic [40]. The pathogenesis and clinical manifestations of ovine babesiosis caused by *B ovis* are well-known, but there is a lack of information on caprine babesiosis [3,39,41,42]. In here, clinical, parasitological, hemotological and serum biochemical data from immunosuppressed and immunocompetent purebred Saanen goats were assessed to create a clear picture of the pathogenicity and virulence of newly identified *B*. *aktasi*. Our findings indicated that the novel *B*. *aktasi* was highly pathogenic to both immunosuppressed and immunocompetent purebred Saanen goats.

Studies on host resistance against bovine *Babesia* spp. have shown that host genotype plays a significant role [27,28]. However, detailed information regarding genetic resistance to ovine *Babesia* spp. is not available [29]. In a previous study carried out by Malandrin et al. [43], *B*. *divergens* infection model in sheep was established. The study indicated that the sensitivity of sheep erythrocytes to *B*. *divergens* varied among different sheep breeds, and concluded that if erythrocytes were resistant to this parasite *in vitro*, it was difficult for sheep to become infected with *B*. *divergens*. An *in vitro* study conducted in China demonstrated that the Chinese Tan mutton breed sheep erythrocytes were highly sensitive to *Babesia* sp. BQ1 (Lintan), while French Vendéen breed sheep were not susceptible to the parasite [29]. These findings suggest that different breeds of the same host species may vary in their susceptibility to *Babesia* parasites. In our previous study investigating the pathogenicity of *B*. *aktasi* in indigenous goats [20], the parasite caused mild fever but did not induce clinical infection in immunocompetent goats. However, it is not known whether *B*. *aktasi* will exhibit different behavior in terms of pathogenicity and virulence in purebred Saanen goats. In the current experimental study using fresh blood infected with *B*. *aktasi*, this question was addressed in purebred Saanen goats. The findings obtained in this experiment indicated that *B*. *aktasi* exhibited variable pathogenic effect among different breeds of the same host species, and induced severe clinical infections resulting in death in purebred Saanen goats.

Experimental studies indicated that the spleen plays a significant role in host defense against *Babesia* spp. After recovering of acute babesiosis, PPE rapidly decreases, but ^th^e parasite persists in the host for an extended period (even at levels undetectable under the microscope). When the spleen is removed in these carrier hosts, PPE increases, and clinical infection occurs, leading to the death of the host [11,19,44]. This situation allows for the *in vivo* isolation of *Babesia* parasites and facilitates its pathogenicity studies. It has been reported that ovine *Babesia* spp. have different pathogenicity and exhibit variations in their response to splenectomy. Experimental studies with *B*. *ovis* demonstrated that all splenectomized sheep died due to severe clinical infection [45,46]. On the other hand, experimental studies with *B*. *motasi* and *B*. *crassa* demonstrated that splenectomized sheep did not develop clinical infection resulting in mortality [47–48]. When blood obtained from a naturally infected sheep with *Babesia* sp. BQ1 (Ningxian) was inoculated into two sheep, one with splenectomized and the other with spleen-intact, the splenectomized sheep exhibited severe clinical symptoms resulting in death including an increase temperature to 41.5°C. In the same study, similar findings were observed in the spleen-intact sheep, but the sheep recovered from the disease [49]. In our previous study, serious clinical infection resulting in death was observed in immunosuppressed indigenous breed goats infected with *B*. *aktasi* [20]. Furthermore, it has been reported that in experimental infections conducted on immunosuppressed sheeps, both clinical and microscopic, as well as molecular examinations, indicated that it did not cause infection [50]. In the current study, serious clinical infection was also occurred in immunosuppressed Saanen goats, and four out of five goats died on the 4^th^ day post-fresh infected blood injection due to severe clinical symptoms, while one goat died on the 7^th^ day.

It has been reported that splenectomy and dexamethasone are the most suitable approaches for the experimental studies conducted on *Babesia ovata*, *Babesia bigemina* and *Babesia* sp. Xinjiang to suppress the immune system [11,51,52]. In an experimental study conducted in China, splenectomized sheep infected with *Babesia* sp. Xinjiang survived acute infection, but death occurred in the sheep that received splenectomy combined with dexamethasone [11]. In an experimental study with *Babesia* sp. BQ1 (Lintan), it was reported that clinical infection did not develop in splenectomized goats. However, when dexamethasone was injected to the same goats, clinical signs of the disease and a high PPE reaching up to 85% were observed [53]. In a subsequent experimental study using the same isolate, *Babesia* sp. BQ1 (Lintan) did not induce clinical infection in spleen-intact sheep and goats [54]. In our previous study, dexamethasone was injected to the splenectomized indigenous goats to fully reveal the effects of *B*. *aktasi*, and this combined approach increased the occurrence of clinical signs of babesiosis [20]. It is not known whether *B*. *aktasi* has a pathogenic effect on purebred Saanen goats. Therefore, in the current study, dexamethasone was injected to the splenectomized goats to achieve complete suppression of the immune system, consistent with previous studies [11,20,51,52,54]. Thus, we obtained more convincing data regarding the pathogenicity and virulence of *B*. *aktasi* on purebred Saanen goats.

In this study, both immunosuppressed and immunocompetent Saanen goats exhibited severe clinical findings such as high fever, anemia, jaundice, and hemoglobinuria. In both groups, the severe clinical infection led to the death of the goats. These findings are consistent with previous experimental studies conducted on lambs infected with *B*. *ovis* [45,46]. *Babesia motasi* Ameland strain did not cause PPE or clinical symptoms in splenectomized goats [48], on the contrary, *B*. *crassa* induced a mild fever and PPE [55]. In our previous study conducted on indigenous goats, *B*. *aktasi* induced severe clinical symptoms in the immune suppressed goats [20]. These findings indicate that different *Babesia* spp. may cause varying clinical symptoms depending on the presence or absence of the spleen [56–59]. It is well-known that distinct *Babesia* spp. can lead to different clinical signs. For instance, *B*. *motasi* Wales strain caused mild fever, anemia, and PPE up to 1% in spleen-intact goats [48]. In our previous study [20], we determined that *B*. *aktasi* has a non-pathogenic effect on the immunocompetent indigenous goats. Interestingly, in the current study, *B*. *aktasi* induced serious clinical babesiosis leading to death in purebred immunocompetent Saanen goats. These results indicated that goat breed plays a vital role in the severity of *B*. *aktasi* infection, and the parasite poses a significant health risk for purebred Saanen goats.

The main hematological measurements considered in the determination of anemia are total RBC, HCT and Hb values. In our study, a statistically significant decrease in HCT, RBC, WBC, and Hb values was observed (Table 2 and Fig 3), and the decrease of HCT, RBC and Hb were inversely associated with the increase in PPE (Figs 4 and 5). Similar results have been obtained in previous studies, suggesting that this decline in the aforementioned blood parameters may be attributed to the destruction of erythrocytes [9,20,60–63]. Our previous experimental study conducted on indigenous goats using *B*. *aktasi* revealed that MCV was normal in both the immunosuppressed and immunocompetent groups, while there was a statistically significant decrease in MCHC value in the immunosuppressed group, indicating a decrease in hemoglobin concentration within RBCs [20]. In contrast to our previous findings [20], the current study observed a significant increase in MCV values and a decrease in MCHC values in the immunocompetent group. Additionally, it was determined that these values remained within the normal range in the immunosuppressed group. These findings indicated that the anemia was macrocytic-hypochromic character in the immunocompetent goats. These findings showed differences from the results obtained in previous studies on *B*. *bovis* and *B*. *bigemina* in cattle [64,65]. A recent study conducted on sheep infected with *B*. *ovis* reported that the RDW (red cell distribution width) value was within the reference range before and after treatment [9]. In the current study, no significant change in RDW-CD and RDW-SD values was detected in the immunosuppressed group, but a significant increase was observed in the spleen intact group (Table 2 and Fig 3). This result indicates a significant reticulocytosis (an increase in young red blood cells) in the spleen intact group, suggesting that the infection lasted longer in this group. Also this finding suggests that spleen function may play an important role in controlling *Babesia* infection [66].

It has been reported that *Babesia* parasites cause cellular destruction resulting in hemolysis in the vascular system, inflammatory lesions and anoxia, particularly in certain organs such as liver and kidneys inducing nephrotoxic effects causing to an elevated AST, ALT, BUN and creatinine levels [9,67]. In this study, along with the increase in PPE, a statistical significant increase in ALT and AST levels was observed in both experimental groups compared to pre-infection levels. Our results agree with the previous studies conducted on indigenous goats experimentally infected with *B*. *aktasi* [20] and sheep naturally infected with *B*. *ovis* [9]. An increase in ALT and AST values indicating hepatic dysfunction has also been observed in cattle infected with *B*. *bovis* [68]. ALT elevation has been previously reported in animals with babesiosis, suggesting that this increase may be due to alterations in liver function associated with the disease [9,69]. Recent studies indicated that certain *Babesia* spp. can cause damage to liver tissue, potentially suppressing the overall liver function [9,69,70].

The pathology of babesiosis is related to the haemolytic anaemia, as a consequence of the intravascular destruction of erythrocytes either by the immune system or by the direct damage caused by the parasite itself [67]. In this study, postmortem changes observed in the goats subjected to necropsy were similar to those previously observed in sheep experimentally infected with *B*. *ovis* [71,72]. Serious pulmonary edema suggest that emphysema may lead to a respiratory failure and could provide direct evidence for death [47,72,73]. Therefore, it can be argued that babesiosis is a respiratory tract disease.

Experimental studies conducted on different goat breeds will allow for a better understanding of the pathogenicity and virulence of *B*. *aktasi*. In contrast, purebred Saanen goats, which have been selectively bred for traits such as milk production, may be more susceptible to infections like *B*. *aktasi*. Studies have shown that while purebred Saanen goats are highly susceptible to trypanosome infections, Saanen crossbreeds exhibit greater resilience [74]. This breed-specific susceptibility underscores the importance of considering breed-specific responses when evaluating the pathogenicity of infectious agents.

In conclusion, in both groups, besides specific signs of acute clinical babesiosis (high fever, anemia, jaundice, hemoglobinuria), general symptoms such as lethargy, anorexia, teeth grinding, trembling, groaning, rapid respiration, lying down with the head on the ground, and immobility were also observed. Shortly after parasite inoculation, PPE was detected at rates ranging from 4.3% to 33.5% in the immunosuppressed group, while it was determined to be 2.1% to 7.6% in the immunocompetent individuals. In the dead goats due to severe clinical babesiosis, edema in the lungs, frothy fluid in the lung and trachea, jaundice in the subcutaneous fat tissue and mesenteric fat, and dark red urine in the urinary bladder were observed. These findings suggest that *B*. *aktasi* is a pathogenic species causing acute infections that result in mortality in purebred Saanen goats. Additionally, considering the abundance of wild goats in the region where the parasite is prevalent, further research is needed to identify the natural reservoirs of *B*. *aktasi*. These further studies will provide valuable information regarding the biology, epidemiology, and ecology of the parasite.
